# Culture and Business: How Can Cultural Psychologists Contribute to Research on Behaviors in the Marketplace and Workplace?

**DOI:** 10.3389/fpsyg.2020.01304

**Published:** 2020-07-15

**Authors:** Takahiko Masuda, Kenichi Ito, Jinju Lee, Satoko Suzuki, Yuto Yasuda, Satoshi Akutsu

**Affiliations:** ^1^Department of Psychology, University of Alberta, Edmonton, AB, Canada; ^2^School of Social Sciences, Nanyang Technological University, Singapore, Singapore; ^3^School of International Corporate Strategy, Hitotsubashi University Business School, Tokyo, Japan

**Keywords:** individualism vs. collectivism, independence vs. interdependence, analytic vs. holistic cognition, vertical vs. horizontal orientation, tightness vs. looseness, strong vs. weak uncertainty avoidance, consumer psychology, organizational psychology

## Abstract

Cultural psychology has great potential to expand its research frameworks to more applied research fields in business such as marketing and organizational studies while going beyond basic psychological processes to more complex social practices. In fact, the number of cross-cultural business studies has grown constantly over the past 20 years. Nonetheless, the theoretical and methodological closeness between cultural psychology and these business-oriented studies has not been fully recognized by scholars in cultural psychology. In this paper, we briefly introduce six representative cultural constructs commonly applied in business research, which include *(1) individualism vs. collectivism, (2) independence vs. interdependence, (3) analytic vs. holistic cognition, (4) vertical vs. horizontal orientation, (5) tightness vs. looseness*, and (6) *strong vs. weak uncertainty avoidance.* We plot the constructs on a chart to conceptually represent a common ground between cultural psychology and business research. We then review some representative empirical studies from the research fields of marketing and organizational studies which utilize at least one of these six constructs in their research frameworks. At the end of the paper, we recommend some future directions for further advancing collaboration with scholars in the field of marketing and organizational studies, while referring to theoretical and methodological issues.

## Introduction

For the past 30 years, the field of cultural psychology has succeeded in addressing the diversity of human psychological processes such as cognition, emotion, and motivation, aiming to demonstrate the substantial effects of culture on basic psychological processes and advocating the need to include cultural theories in mainstream psychological academics. However, until recently, cultural psychologists’ motivations have been to develop behavioral as well as neuroscientific measures (Kitayama and Uskul, 2011) and their studies have focused on so-called basic processes such as perception, cognition, emotion, and motivation, thus they have often failed to investigate possible cultural differences when analyzing concrete social practices.

Meanwhile, scholars in the field of marketing and organizational behavior have focused on a variety of topics which are very much relevant to what cultural psychologists have thought of as examples of social practices. These scholars accumulate evidence of diversity in our daily activities, notably our behaviors in the marketplace as buyers and sellers, and our behaviors in the workplace as employers and employees. They also seek answers to concrete questions such as, “How do we select products at a supermarket for tonight’s dinner?” “How do we purchase a present for our significant other?” “How do we select a job?” “What factors direct us to leave a job?” “How do we organize a business team?” and “How does a company create a positive image?”

Indeed, the number of publications referring to business culture has increased exponentially. For example, in their review paper published in the Journal of Applied Psychology celebrating the journal’s anniversary, Gelfand et al. (2017) summarized the past 100 years of trends in cross-cultural research in industrial and organizational psychology, explaining how culture has gradually come to play a role in this field. Similarly, in their review paper in Consumer Psychology Review, Shavitt and Barnes (2018) mentioned that the field of consumer psychology is maturing and that researchers are increasingly recognizing culture as an important factor in the study of business.

However, scholars who identify cultural psychology as their main research domain have not fully recognized the theoretical and methodological closeness between cultural psychology and these business-oriented studies. It is important for them to better understand this possible affinity and to be exposed to what business-oriented studies have done for the past 20 years. We maintain that exposure will strengthen the relationship between these two research areas while further advancing both scientific fields. How can cultural psychologists increase their contributions to business research?

To answer this question, this conceptual paper reviews six representative, measurable cultural constructs as tools from social and cultural psychology which either have already gained traction in or that we believe could be extremely useful to fields of business research: (1) *individualism vs. collectivism*, (2) *independence vs. interdependence*, (3) *analytic vs. holistic cognition*, (4) *vertical vs. horizontal orientation*, (5) *tightness vs. looseness*, and (6) *strong vs. weak uncertainty avoidance*. We then review some representative studies from two major fields of business research, marketing and organizational behavior, after which we discuss the natural affinity between cultural psychology and business research. Finally, we address some theoretical and methodological issues to help cultural psychologists find purchase in areas of applied business research in order to contribute to and develop the field.

## Emergence of Theoretical Frameworks and Methodology for Cross-Cultural Research

The seeds of culture-based investigation of human behavior trace back to a few psychological and sociological works such as Wilhelm Wundt’s (1916) “Elements of folk-psychology” and Max Weber (1905/1958)’s “The Protestant Ethic and the Spirit of Capitalism.” However, extensive cross-cultural empirical investigations in the modern era did not appear until the 1980s, beginning with Geert Hofstede’s *cultural dimensions theory* introduced in his book published in 1980. In this section, we introduce six major theoretical frameworks which were devised over the past 40 years in the fields of social and cultural psychology. We will refer to these dichotomous theoretical frameworks as “cultural constructs.” As we will see in the later sections, these constructs have been most highly adopted by business researchers for analyses of marketing and organizational behaviors. Therefore, we consider them to be fundamental starting points for collaboration between cultural psychology and business researchers. Now, we will briefly introduce the scope of each cultural construct.

### Individualism vs. Collectivism Construct

This construct describes people’s preference to act as individuals or in groups in society. Geert Hofstede (1980) introduced this construct in his *cultural dimensions theory* as one of four dichotomies he extrapolated from an extensive analysis of global IBM employees’ cross-cultural data. The dimension of individualism and collectivism differentiates people’s values in the workplace. Hofstede (1980) also introduced three other dimensions: (2) *small* vs. *large power distance* (acceptance of power inequality in organizations), (3) *strong* vs. *weak uncertainty avoidance* (the degree of tolerance to ambiguity and uncertainty about the future), and (4) *masculinity* vs. *femininity* (preference for achievement and assertiveness vs. modesty and a nurturing relationship). Later, Hofstede and Bond (1988), Hofstede et al. (2010), and Hofstede (2011) added two more dimensions to the theory: (5) *short-* vs. *long-term orientation* (flexible vs. fixed view toward the world) and (6) *indulgence* vs. *restraint* (valuing freedom vs. valuing duty).

Of these six dimensions, *individualism vs. collectivism* has particularly caught business scholars’ interest and has gained popularity in cross-cultural business research, partly because it explains the divisions that are popular across the social sciences and also because it is extremely useful for analyses of group behaviors in any setting. The dichotomy has been adapted to be applied at the level of the individual; Harry Triandis’ group has numerous publications that address *individualism vs. collectivism* as an indispensable tool with which to assess cultural variations in human values.

### Independence vs. Interdependence Construct

Markus and Kitayama (1991, 2010) extend the social psychological investigation of the self and suggest that cultural diversity displayed in basic psychological processes such as cognition, emotion, and motivation can be mapped against one’s self-construal. Those who live in a culture where the *independent self-construal* is dominant tend to view themselves as being separate from social others and hold cognitive styles that emphasize self-direction, autonomy, and self-expression. By contrast, those who live in a culture where the *interdependent self-construal* is dominant tend to view themselves as socially interrelated and connected to significant relationships and hold cognitive styles that emphasize harmony, relatedness, and connection. This construct’s self-report scales allow researchers to assess an individual’s concept of identity (Singelis, 1994; Singelis and Brown, 1995; Kim et al., 2003). The concept of *independent vs. interdependent self-construal* has become popular among researchers in marketing and organizational psychology (e.g., Erez and Earley, 1993) because of its focus on self-identity in the marketplace and workplace.

### Analytic vs. Holistic Cognition Construct

Richard Nisbett and his colleagues approach cultural aspects of cognition by reviewing the historical origins of Western and Eastern thinking styles. Working under the construct of *analytic vs. holistic cognition*, they maintain that culturally and historically shared and sustained thinking styles shape even our basic psychological processes such as perception and cognition (Peng and Nisbett, 1999; Nisbett et al., 2001; Nisbett, 2003; Nisbett and Masuda, 2003; Nisbett and Miyamoto, 2005). *Analytic cognition*, dominant in Western cultures such as Western Europe and North America, is characterized by discourse that emphasizes an object-oriented focus in visual attention (selectively focusing more on objects than on context). In contrast, *holistic cognition*, dominant in East Asian cultures such as China, South Korea, and Japan, is characterized by discourse that emphasizes a context-oriented focus of attention (attending to objects in relation to their context). Two useful self-report scales are available under this construct: a dialectical self-scale (Spencer-Rodgers et al., unpublished) and a holism scale (Choi et al., 2007), which allow scholars to assess the associations between the score and dependent variables. However, other scholars conduct studies by creating cognitive and perceptual tasks (see Masuda et al., 2019 for a review). We believe that this construct may be valuable to marketing studies because it can assess how a target audience responds to visual media as well as their range of attention to others during interpersonal interactions.

### Vertical vs. Horizontal Orientation Construct

Harry Triandis’s group (e.g., Triandis, 1989; Triandis and Gelfand, 1998) suggests that there is another useful dichotomy to typologically categorize human values globally: *vertical vs. horizontal*. Vertically oriented individuals seek status and authority, while horizontally oriented individuals prefer equality. This dichotomy contrasts well with the aforementioned *individualism vs. collectivism* dichotomy, providing an additional axis to map societal characteristics into four quadrants. A battery of self-report scales has been developed to plot data across the two dimensions, allowing researchers to finely detect cultural variability in human values (e.g., Triandis and Gelfand, 1998). Given the hierarchies of both culture and business, this construct should prove to be broadly beneficial. Research under this construct has determined representative societies for each quadrant based on a corresponding scoring item: *vertical individualism*—“It is important that I do my job better than others:” the United States, the United Kingdom, and France; *vertical collectivism*—“It is important to me that I respect the decisions made by my groups:” Japan, South Korea, and India; *horizontal individualism*—“I’d rather depend on myself than others:” Sweden, Norway, and Australia; and *horizontal collectivism*—“To me, pleasure is spending time with others:” Brazil. In a later section, we will discuss the similarity of scope between Hofstede’s *power distance* and Triandis’ *vertical* vs. *horizontal orientation*.

### Tightness vs. Looseness Construct

The dichotomy of *tightness* vs. *looseness culture* advocated by Michele Gelfand and her colleagues is the latest cultural construct to have been introduced, filling a gap in cross-cultural investigation of human behaviors (Gelfand et al., 2011; Harrington and Gelfand, 2014; Mu et al., 2015; Gelfand, 2018). They define *tight cultures* as social systems in which social norms are clearly defined and less tolerant of deviant behaviors. Examples of tight cultures include China, Germany, Mexico, France, India, Japan, and Singapore. In contrast, *loose cultures* are defined as social systems in which social norms are flexible, informal, and more tolerant of deviant behaviors and include Hungary, Brazil, Australia, Belgium, Israel, New Zealand, and the United States. The level of *looseness vs. tightness orientation* has also been documented as a form of intra-cultural variation. For example, within the United States, Southern regions are considered the tightest, while Western and Northwestern regions are considered the loosest (Aktas et al., 2016). Because companies also exhibit and place value on “tight” and “loose” characteristics, this construct can easily be adapted for use in business research. In a later section, we will describe a study which compares Amazon and Whole Foods under this construct.

### Strong vs. Weak Uncertainty Avoidance Construct

One final dimension of Hofstede’s (1980) early work that we would like to address is uncertainty avoidance, which refers to how a society deals with the fact that the future is unpredictable and whether the society tries to control the future or simply accept it. While this temporal dimension, which is associated with past, present, and future, has the potential to scrutinize diversity in cultural patterns, it has not been widely utilized. It was adapted for use at the individual level of analysis in the early 2010s, and a self-report scale with five items was devised for cross-cultural investigation (Yoo et al., 2011). Since then, it has since gained popularity.

According to the previous findings, Asian countries are similar in their level of collectivism, but Hofstede’s dataset (Hofstede et al., 2010) showed that there are surprisingly substantial differences between Asian societies’ tolerances for uncertainty, as shown by their scores for uncertainty avoidance. Hong Kong (29 points), Singapore (8), and China (30) demonstrated low scores for uncertainty avoidance, suggesting that these countries hold relaxed attitudes toward their futures, while South Korea (83) and Japan (92) scored high, suggesting that they hold more rigid attitudes toward uncertainty and seek a sense of control regarding their futures. Similarly, of the so-called individualistic Western societies, France (86), Spain (86), and Italy (75) displayed high uncertainty avoidance, while the United States (46), Canada (48), and the United Kingdom (35) scored low. These results make us question previous claims that “individualistic” Western societies are monolithic; rather, they suggest that there are important nuances that need to be investigated further, and the same goes for our impression of “collectivistic” East Asian societies.

We believe uncertainty avoidance, one of Hofstede’s (1980) original dimensions, deserves special attention because it has relevance to many marketing and organizational phenomena such as innovation, advertising, and financial investments. For instance, differences in the level of uncertainty avoidance in a culture may predict a tendency for advertising in that culture to appeal to fear or safety.

## Relationships Between Past and Current Constructs

In this section, we discuss the relationships between the six constructs described above and their development from predecessor constructs which emerged from earlier studies and essays. Some of the constructs are conceptually similar to each other, but researchers have treated some of them as conceptually distinct. We will attempt to clarify the value of these constructs and explain how each has been applied to individual-level analyses by introducing some representative publications. We believe that this assessment will facilitate better understanding of the characteristics and use case for each construct. It is our hope that describing them in detail together here will prove useful not only to psychologists but also to business scholars and will foster increased interdisciplinary collaboration.

**FIGURE 1:**
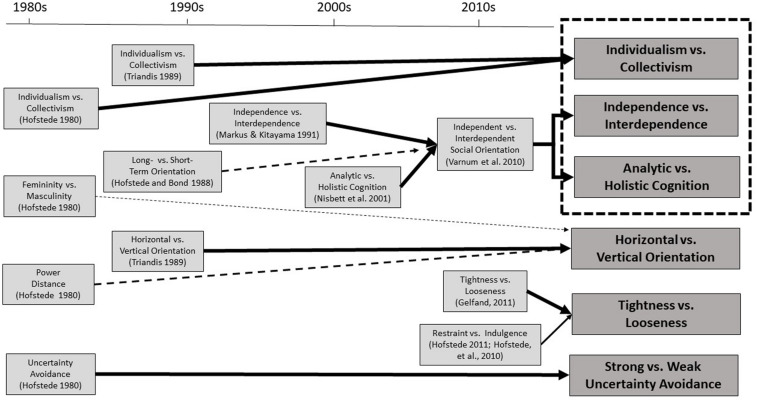
Schematic figure showing chronology and relationships between past and present cultural constructs.

Figure 1, below, presents the target cultural constructs chronologically, starting from Hofstede’s (1980) original four dimensions (*individualism vs. collectivism, masculinity vs. femininity, power distance, and uncertainty avoidance*). *Short- vs. long-term orientation* was introduced slightly later (Hofstede and Bond, 1988). Triandis (1989) introduced two constructs: first, *individualism vs. collectivism*, and at the end of the 1980s, *vertical vs. horizontal orientation*. Seminal papers by Markus and Kitayama were published in 1991 and by Nisbett et al. (2001). The 2010s brought Hofstede et al.’s (2010), Hofstede (2011) *indulgence vs. restraint*, Gelfand et al.’s (2011) *tightness vs. looseness*, and Varnum et al.’s (2010) *social orientation theory*. The style of line represents the conceptual relationship between the constructs: bold for a strong or direct relationship, wide dotted for a intermediate relationship, and narrow dotted for a weak relationship. Finally, the dotted rectangle in the top right corner indicates that the top three cultural constructs are often categorized as a single construct in business research, especially in marketing studies (e.g., Shavitt and Barnes, 2018), as we will discuss later.

### Independent vs. Interdependent Social Orientation

As noted in the previous section, theoretical constructs evolve over time and are used to create mainstream tools. Varnum et al. (2010) noted the similarities between the *independence vs. interdependence* and *analytic vs. holistic* constructs and merged them under the name of *independent vs. interdependent social orientation* (Figure 1, top-middle). People who live in a society where independent social orientation is dominant are likely to define themselves based on their personal abilities, talents, propensities, and traits, and they expect that other people also have such internal attributes. They are more attentive to personal factors and develop a social reality in which society is the sum of independent individuals, resulting in an analytic worldview in which they understand the world by identifying the stable essence of each individual element.

In contrast, people who live in a society in which the interdependent social orientation is dominant are expected to accommodate to societal requirements and develop skills to meet societal standards. To meet those standards, they become more attentive in searching for outside information and develop a holistic social reality of living in a complex system, resulting in acceptance of a worldview in which everything is interconnected.

However, some scholars have recently claimed that these two constructs are complementary, and shed light on different levels of culturally unique psychological processes (see also Miyamoto, 2013; Masuda et al., 2019; for further discussion). For example, Miyamoto (2013) maintains that *social orientation* is a higher-level construct, with *analytic vs. holistic cognition* as a sub-category. She claims that *analytic vs. holistic cognition* better analyzes an individual’s perceptual and cognitive domains such as object-oriented attention vs. context-oriented attention.

In contrast, *independence vs. interdependence* better analyzes an individual’s domains of social cognition such as socially disengaging emotion vs. socially engaging emotion, self-enhancing motivation vs. self-critical motivation, and trait-based self-perception vs. role-based self-perception. This paper assumes that there are nuanced differences in the targets of analyses and therefore keeps *independent vs. interdependent* and *analytic vs. holistic* cognition separate from each other in Figure 1. In a later section, we will review research on the marketplace and workplace that utilizes these two mutually relevant but independent constructs.

### Individualism vs. Collectivism Construct and Social Orientation Construct

As stated previously, of all the constructs introduced in Hofstede’s (1980) cultural dimensions theory, the *individualism vs. collectivism* dimension turned out to be the most popular over the past four decades, and a variety of cross-cultural studies have been conducted based on this construct. However, the construct we are presenting in this paper is not identical to Hofstede’s original concept but rather represents a convergence of ideas that have been developed and refined over time, informed by research by Triandis (1989) and others (Figure 1, top-right).

In fact, researchers often consider these three constructs as a single construct under a larger umbrella (labels arbitrarily depend on the research group’s emphasis) (Figure 1, box with dotted border), and the names of the constructs are often referred to interchangeably within a single text. For example, in their conceptual paper about the importance of cross-cultural research on consumer behaviors, Shavitt and Barnes (2018) merged these three constructs into one. Other cross-cultural studies which focus on Easterners and Westerners have given credence to their assertions: overall, results of studies under these constructs converge, demonstrating that Easterners such as Chinese, Japanese, and Koreans tend to hold collectivistic, interdependent, and holistic mentalities, whereas Westerners such as Americans, Canadians, West Europeans, New Zealanders, and Australians tend to hold individualistic, independent, and analytic mentalities.

Nevertheless, several studies which targeted cultural groups outside the East vs. West dichotomy provide evidence that holistic tendencies should be treated as independent from interdependent-self construals. For example, Spencer-Rodgers et al. (2004) demonstrate that while Latinos and African Americans have been reported to be interdependent/collectivistic in general, their holistic tendencies are lower than those of European Americans’. Theoretically, we could imagine a culture which is high in collectivism/interdependence and high in analytic cognition is possible. However, to date, there are no studies which report such societies or groups, and thus it may be advisable to assume that holism emerged in ancient China has only spread to other East Asian societies (e.g., Nisbett, 2003). Therefore, we argue that *independence vs. interdependence* and *analytic vs. holistic* should be considered as separate constructs pending further studies (Figure 1, split arrow). Extending research into lesser-studied societies, such as Mongolia and South American indigenous groups, may allow us to better refine our understanding of the relationship between these two constructs.

### Vertical vs. Horizontal Orientation and Power Distance Dimension

The issue of power in cultural contexts has long been discussed in social psychology. McClelland (1973) discussed two types of power-relevant motives: personalized motives, which emphasize the powerholder’s self-interests, and socialized motives which emphasize benefits to others. In the cross-cultural context, Hofstede (1980, 2011) introduced the *power distance dimension* as one of his cultural dimensions. This dimension deals with the issue of how people perceive inequality in a given society. A large power distance refers to a case in which employees tend to accept an existing hierarchical order and power inequality, whereas a small power distance refers to a case where employees strive to equalize the power distribution and seek justifications for inequalities of power. In a similar vein, Triandis (1989) describes the concept of power as an individual’s sense of competitiveness and self-centeredness under the name *vertical vs. horizontal dimension*. As such, the issue of power can be conceptualized both from powerholders’ and non-powerholders’ points of view.

Recently, Torelli and Shavitt (2010) addressed culturally nurtured views of power in which they redefine the target of analysis using Triandis (1989) combination of *individualism vs. collectivism* and *vertical vs. horizontal dimension.* Here, we go over the definition of these combinations and explain how power is conceptualized in each one. Subjects high in *vertical individualism (VI)* emphasize self-achievement and pay less attention to others’ benefits; they are selectively focused on personalized power rather than socialized power. Those high in *horizontal individualism (HI)* put less emphasis on their own goals and pay less attention to power-related issues such as achieving status and helping others; they have low motivation for both personalized and socialized power. Those high in *horizontal collectivism (HC)* emphasize socialized use of power for helping others and are against personalized power and authorities who abuse their power. Those high in *vertical collectivism (VC)* show two identifiably different patterns depending on whether they can access power: high powerholders feel a strong duty to endorse power for their in-group members, whereas low powerholders are less likely to hold such motives.

Shavitt et al. (2006) and Torelli and Shavitt (2010) have attempted to reconsider the relationship between culture and power, but recognize the substantial differences between how previous research has interpreted the cultural constructs both methodologically and conceptually, and encourage researchers to try to further synthesize existing cultural constructs under one umbrella. In this paper, we propose that some past constructs could be resynthesized in a modern way. For example, although Hofstede’s (1980, 2010) *masculinity vs. femininity dimension* has been treated independently from other dimensions, in its broadest definition, it obviously touches on issues of power, competitiveness, and status. However, it has not been comprehensively utilized in the context of culture. With further research, it may be possible to merge this dimension into the framework of the *vertical vs. horizontal* construct (Figure 1, middle converging arrows).

### Hofstede’s Influence on Looseness vs. Tightness Constructs

Hofstede et al.’s (2010), Hofstede (2011) *indulgence vs. restraint* dimension was introduced relatively late and has retained minor status in his cultural dimension model (Figure 1, bottom-right). *Indulgent* societies allow relatively free gratification of basic and natural human drives related to enjoying life and having fun. According to Hofstede et al.’s (2010) dataset, Canada (68 points), the United States (68), and the United Kingdom (69) are some representative societies high in *indulgence.* In contrast, *restraint* indicates that a society suppresses gratification of needs and regulates them by means of strict social norms. East Asian societies such as Hong Kong (17), China (24), and South Korea (29) are examples of societies high in *restraint*, followed by Japan (42) and Singapore (46). Although few studies have examined the association between Hofstede’s *indulgence vs. restraint dimension* and Gelfand et al.’s (2011) and Gelfand et al. (2018) *tightness vs. looseness* construct, we maintain that they have a strong association and that Hofstede’s dimension might be better redefined as a subcategory of *tightness vs. looseness* (Figure 1, arrow between the two constructs).

### The Role of *Long- vs. Short-Term Orientation* in *Analytic vs. Holistic Cognition*

There is one more dimension which we have not yet mentioned: *long- vs. short-term orientation* from Hofstede and Bond (1988). They sought out little-used concepts which had been dropped from their original theories, and were especially motivated to develop a dimension based on Chinese local values. Societies with long-term orientation emphasize persistence, personal adaptability, and a spectrum between good and evil, whereas short-term orientated societies emphasize quick outcomes, personal steadfastness and stability, and a binary view toward good and evil. In Hofstede et al.’s (2010) dataset, many East Asian countries such as China (87 points), Japan (88), and South Korea (100) score high in long-term orientation, followed by Singapore (72) and Hong Kong (61). North American countries such as Canada (36) and the United States (26) score high in short-term orientation. Although the relationship between this dimension and *analytic vs. holistic* cognition has not been extensively explored yet, we suggest considering a strong association between them. In fact, in a new line of research on temporal aspects of *analytic vs. holistic* cognition, Ji et al. (2009) demonstrated that East Asians hold a long-term perspective whereas North Americans hold a short-term perspective about future events. While further theoretical elaboration is required, this paper treats Hofstede and Bond’s dimension as a sub-category under the *analytic vs. holistic* cognition umbrella (see the arrow between the two constructs in Figure 1).

### Summary

This section introduced six cultural constructs which have been commonly used in business research and which can facilitate cultural psychologists to collaborate in that field. To better capture the characteristics of the six listed constructs, we discussed the relationships between constructs generated by different academic groups in order to provide a “tentatively integrated big picture” of cultural constructs. Of course, there are many other popular constructs, for example, *allocentrism–idiocentrism* (Triandis et al., 1985), Schwartz’s *structure of human values* (Schwartz and Bilsky, 1990; Schwartz, 1992, 1994), *prevention-focus* vs. *promotion-focus of motivation* (Higgins, 1997; Higgins et al., 2001), and *incremental* vs. *entity theory* (Dweck et al., 1993, 1995), which enable scholars to cross-culturally test patterns of behaviors in the marketplace and workplace. However, it is too early to comprehensively sort out and nest all the available constructs into a hierarchy, and it is beyond the scope of this paper. Let us just suggest that there remains much work that can be done.

Before closing this section, there is one issue to be addressed, and that is the level of analysis. Although Hofstede’s framework was originally developed at the national/country level, as the areas of research matured, and empirical studies accumulated, many scholars narrowed its application to the level of the individual, as well. In fact, in Kirkman et al.’s (2006) review of 180 studies published in business and psychology journals, they found that 48% adapted Hofstede’s framework to an individual level. Of the remaining studies, 46% used the framework at the country level, and 6% adapted it to a group/organizational level.

Hence, there are substantial differences in conceptualization and application of cultural constructs. On one side, scholars maintain national-level analyses. For example, Brewer and Venaik (2014) raised concern that Hofstede’s framework has been incorrectly applied in studies which target individual- and organizational-level analyses, calling it “an ecological fallacy in national culture research.” They further suggested that a cautious methodological approach is warranted in order to avoid inferring individual or organizational behaviors from aggregated national data. On the other side, Devinney and Hohberger (2017) argue that the manifestation of national culture can be observed at multiple levels; therefore, they request substantial modification to Hofstede’s framework. While the debate is worthwhile, this paper conceptualizes cross-cultural research utilizing cultural constructs at the individual level because the majority of studies we will review next are based on individual-level analyses measuring psychological and behavioral phenomena within their cultural frameworks.

## Cross-Cultural Investigations of Human Behaviors in the Marketplace and Workplace

**FIGURE 2:**
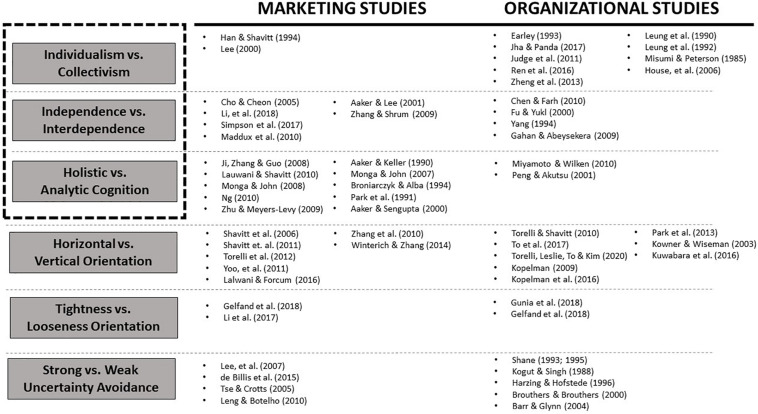
Representative works in marketing and organizational studies.

In this section, we review some representative empirical studies from the research fields of marketing and organizational studies which apply at least one of these six constructs to their research framework. We have chosen to focus on these two areas because they are the areas of business research most connected to the study of culture. Of course, they are not the only areas in which culture research is valuable, but the number of available studies makes them a good starting point (see Figure 2).

## Marketing Studies Relating to Culture

Marketing is a major area in business schools which investigates human behaviors in the marketplace such as consumer behavior, advertising, and branding strategies. The importance of culture in marketing studies has been recognized since the early 2000s, and now, after 20 years, the cultural construct-based approach is flourishing in this area (Maheswaran and Shavitt, 2000; Shavitt et al., 2008; Shavitt and Barnes, 2018). In this section, we briefly review classic and recent findings in the area of marketing conducted under the rubric of cultural constructs.

### Research Utilizing the Individualism vs. Collectivism Construct

This popular construct has been used in numerous studies investigating a variety of themes. Below, we review some compelling studies in areas that may be of interest to cultural psychologists looking for opportunities for collaboration.

#### Consumer Persuasion

Han and Shavitt’s (1994) study was one of the first cross-cultural works and claimed that American ads tend to emphasize individual benefit and preference, personal success, and independence, whereas Korean ads tend to emphasize in-group benefit, harmony, and family integrity. Furthermore, they demonstrated that the contents of ads correspond to one’s mentality regarding persuasiveness: Americans indeed found it persuasive when ads emphasized individualism, while Koreans found it persuasive when ads emphasized collectivism. As subject matter experts in culture, cognition, and research methodology, cultural psychologists could contribute greatly to the studies of consumer persuasion with their analyses.

#### Consumer Intention

Lee’s (2000) survey research was one of the first cross-cultural works to actively advocate the importance of this construct in marketing research. This cross-cultural survey was conducted in collectivist countries (Singapore, Hong Kong, and South Korea) and individualistic countries (Australia and the United States) and investigated participants’ purchase intentions, consequences, and attitudes toward target products, while testing the overall fit for their proposed model, which attempted to depict the relationships between product purchase and attitude-intention-context-behaviors. While the results indicated a high degree of fit for her model, Lee (2000) reported cultural variations in what were considered critical factors for the subjects’ purchase decisions: people in collectivist cultures emphasize significant others’ responses to their purchases whereas people in individualistic cultures emphasized their own attitudes for their purchase decisions, suggesting that the key component of attitude may depend on the subject’s culture. Furthermore, cross-cultural variations in the importance of past experiences were also reported: compared to individualists, collectivists tend to be motivated to continue to purchase their society’s well-known products, even when lower-priced products of equal quality become available on the market. These findings may set the foundation for future research on consumer behaviors. As such, Hofstede’s and Triandis’ models receive a certain recognition in marketing scholars’ discourse and will hopefully attract even more scholars to initiate research using cultural constructs.

### Research Utilizing the Independence vs. Interdependence Construct

As mentioned previously, marketing scholars have adopted independent vs. interdependent self-construals advocated by Markus and Kitayama (1991) into their discourse. Heine (2012) anecdotally reported that independence vs. interdependence influences online market companies’ customer services. eBay, one of the most famous online businesses, launched in San Jose, California, successfully grew to be a worldwide business in over 30 countries. Their attempt to expand into China, however, did not succeed, and they ended up withdrawing from the Chinese market in 2006 only 2 years after entering the market. In contrast, TaoBao, a Chinese domestic online market company, successfully established itself in the Chinese market.

Scholars explored the reasons behind eBay’s failure and TaoBao’s success. Some scholars maintain that the critical difference was their differing approach with regard to the relationship between sellers and buyers (Lafevre, 2013). While eBay emphasized customer services devised for the independent, transactional business culture of North America, China’s interdependent culture emphasizes close interpersonal connections called “guanxi”—a deep structure of reciprocity and mutual obligation between two parties. Indeed, Taobao provided buyers and sellers with a variety of means to get to know each other before purchase and a way to continue to develop long-term relationships after purchase. They also introduced the concept of “team buying,” where customers can receive discounts for buying in quantity as a group. Buyers will expend significant energy creating large social groups in order to maximize group benefit.

Along this line of investigation, Cho and Cheon (2005) reported that East Asian websites were furnished with social media functions which emphasize interactions among buyers, such as creating user groups and communities, whereas Western websites focused on problem resolution and ignored relationship building.

#### Independence vs. Interdependence

Independence vs. interdependence has been the most popular construct used for cross-cultural marketing studies. Other topics with which this construct has been used include cultural variation with regard to the effects of relationships, endowment effects, participants’ prevention vs. promotion tendencies, sense of self-consistency, and impulsiveness. With the rise of online shopping and social media, this construct has a lot of exciting potential.

#### Self-Construals and Effects of Relationships

One of the key elements of interdependence is a sensitivity to relationships with significant others. For instance, in the context of branding strategies, those high in interdependence were more likely than their independent counterparts to be attentive to relational brand characteristics rather than personal brand characteristics (Li et al., 2018). In the context of helping behaviors, those high in interdependence were likely to donate more when their gift was known publicly rather than kept private (Simpson et al., 2017), suggesting they are concerned about how they will be evaluated by others around them and will act to achieve perceived recognition from others.

#### Self-Construals and Endowment Effects

Other scholars examine cultural diversity in one’s sense of ownership, assuming that the level of endowment effect—the tendency to value things they possess rather than things they do not possess—is stronger for those who display high independence than for those who display interdependence. An independent individual strongly values the self. Therefore, once an object is associated with his or her own self-worth, the object is also seen as being more valuable than the same object not of their possession.

For instance, Maddux et al. (2010) demonstrated that endowment was stronger for Westerners and those who were primed with independent self-construals than for East Asians and those who were primed with interdependent self-construals. Gobel et al. (2014) further investigated the boundary condition of the endowment effect, by demonstrating that interdependent Malaysians showed endowment effects in private conditions but not in public conditions, whereas independent British participants showed such effects in both conditions, suggesting that Malaysians’ sense of interdependence attenuated only when their sense of personal possession was activated. Recently, Collard et al. (2020) further demonstrated that there is a strong association between self-perception, endowment effects, and ownership effects—the tendency to enhance one’s memory of owned items by increasing attentional processes.

#### Self-Construals and Impulsiveness

Impulsive purchase behavior has been investigated using this construct. Studies have demonstrated that interdependence rather than independence facilitates people (1) to be less impulsive in consuming goods (Zhang and Shrum, 2009); (2) to plan ahead by purchasing coupons for repeat purchases (Lalwani and Wang, 2018); (3) to be more sensitive to cognitive information than affective information of products and services when they make a purchase (Hong and Chang, 2015).

#### Subcategories of Independence vs. Interdependence

Several scholars have sought to discover dichotomous constructs which could be sub-categories of the independence vs. interdependence construct. For instance, Aaker and colleagues demonstrated that, compared to those with high independence, those with high interdependence were more prevention-oriented than promotion-oriented because they emphasized group harmony and a sense of accommodation rather than assertively seeking personal gain (Lee et al., 2000; Aaker and Lee, 2001; Lee and Aaker, 2004; Agrawal and Maheswaran, 2005; Wang and Lee, 2006).

### Research Utilizing the Holistic vs. Analytic Cognition Construct

Since the publication of Nisbett et al. (2001) and Nisbett’s (2003) work, both cross-cultural and business researchers have demonstrated diversity in cognition and perception in the marketplace related to holism and dialecticism.

#### Holism and Consumer Behaviors

Aaker and Sengupta (2000) reported that when a target product’s source information and attribute information are incongruent, Americans selectively narrow their attention to the attribute information, whereas Hongkongers equally allocate attention to both the source and attribute information. Although Aaker and Sengupta (2000) did not refer directly to holistic vs. analytic cognition, the results echo other studies’ findings on East Asian’s dialectical and holistic thinking styles—leniency to contradiction and holistic reference to all available information—which cultural psychologists reported in later years (e.g., Choi et al., 2003; Li et al., 2016). More studies under the widely accepted modern construct of holistic vs. analytic cognition would be highly valuable additions to the field.

Recent convergent findings report that holistic thinkers are more likely than their analytic counterparts to (1) automatically think that you get what you pay for—perceiving a fixed relationship between the price and the quality of products (Lalwani and Shavitt, 2013), suggesting their bias of holistically searching for covariation among things (Ji et al., 2000); to (2) prefer information about prices of competing brands on the market rather than information about inherent characteristics of the products (Chen, 2009), suggesting attention to external factors rather than internal factors (Morris and Peng, 1994); to (3) expect fluctuations in stock markets rather than linear trends and therefore be inclined to purchase stock when the slope goes down rather than up (Ji et al., 2008) and take into account both positive and negative information about the product rather than selectively focusing on either one or the other (Monga and John, 2007; Ng, 2010), suggesting their holistic yin and yang thinking style.

#### Dialecticism and Consumer Behaviors

Leniency toward rule-based categorizations and contradictions were also counted among some of the important characteristics of holistic thinkers over analytic thinkers (e.g., Nisbett, 2003). In marketing studies, this tendency is observable as holistic thinkers were more likely than their analytic counterparts to (1) evaluate the quality of a target product in relation to context rather than perceive the product as constant across different contexts (Zhu and Meyers-Levy, 2009), suggesting their strong sense of holism, and to (2) make connections between their existing brand attitudes and new brand extensions (Aaker and Keller, 1990; Park et al., 1991; Broniarczyk and Alba, 1994; Monga and John, 2007), showing evidence that they perceive category boundaries loosely (Norenzayan et al., 2002).

### Research Utilizing the Vertical vs. Horizontal Orientation Construct

The number of marketing research papers that refer to *vertical vs. horizontal orientation* has started to increase in the past 10 years. As mentioned before, since a group of scholars introduced this construct and articulated its characteristics relating to the perception of power among cultures demonstrating *vertical individualism* (e.g., the United Kingdom and the United States), *horizontal individualism* (e.g., Norway and Australia), *vertical collectivism* (e.g., Japan and South Korea), and *horizontal collectivism* (e.g., Brazil) (Shavitt et al., 2006; Shavitt and Cho, 2016; Torelli et al., 2017, 2020), scholars are actively delving into psychological processes in the marketplace. For instance, *vertical individualism* is associated with self-centered power, whereas horizontal collectivism is associated with other-centered power (Torelli and Shavitt, 2010). Cognitively, people high in vertical individualism use their power to confirm their initial impression or stereotype of a topic and recognize information of target products congruent with prior information provided, while minimizing their cognitive load. In contrast, people high in horizontal collectivism use their power to better individuate and understand others and recognize information about target products even if it is incongruent with prior information, while not sparing their cognitive load (Torelli and Shavitt, 2011).

Recently, many cross-cultural marketing studies have demonstrated diversity in perception of advertisement and brand images, while addressing the fact that those high in horizontal orientation were more likely than their vertical counterparts to be attentive to personal qualities rather than hierarchy. For instance, those high in horizontal orientation were more likely than their vertical counterparts to (1) create ads which emphasized uniqueness messages (e.g., Denmark) rather than prestige, luxury, and status messages (e.g., South Korea, the United States) (Shavitt et al., 2011) and to (2) enjoy ads which emphasize self-transcendence and openness rather than conservatism and self-enhancement (Torelli et al., 2012). The research on low vs. high power orientation indicated that those high in power orientation were more likely than their low power orientation counterparts to be more favorable to ads featuring celebrity endorsers (Yoo et al., 2011; Winterich et al., 2018), to (1) think that high price corresponds to high quality, because they desire linear order (Lalwani and Forcum, 2016), to (2) engage in less impulsive purchasing (Zhang et al., 2010) because they accept social norms, and to (3) perform fewer charitable behaviors because they accept inequality (Winterich and Zhang, 2014).

### Research Utilizing the Tightness vs. Looseness Construct

The fifth cultural construct which has been applied to marketing research is Gelfand et al.’s (2011) and Gelfand et al. (2018) *tightness vs. looseness* orientation. Since this new construct came to the academy quite recently, the number of studies is few. Nonetheless, the marketing researchers have demonstrated the usefulness of this construct in marketing research. For instance, Li et al. (2017) discussed some possibilities regarding cultural variations in consumer behaviors and advertising and branding strategies.

According to Li et al. (2017), consumers in tight societies, similar to those high in power distance, may develop emotional regulation skills and share a culturally conservative attitude and motivation to abide by rules and norms. If that is the case, the brand image strategies in tight cultures may function well if the ads focus on images about loyalty toward companies and the brand, and the ads facilitate customers to share common ground and consensus regarding the brand. In contrast, brand image strategies in loose cultures may function well if the ads emphasize permissiveness and openness rather than restriction and close-mindedness and if the brand develops a platform for consumers to customize the products to feel a sense of uniqueness.

### Research Utilizing the Strong vs. Weak Uncertainty Avoidance Construct

The last cultural construct, which has been applied to marketing research is *strong vs. weak uncertainty avoidance.* Uncertainty avoidance is defined as “the extent to which people feel threatened by ambiguous situations, and have created beliefs and institutions that try to avoid these” (Hofstede and Bond, 1984, p. 419). It is not difficult to imagine that the majority of our daily decision-making is made without having complete information. Rather, decisions are made under uncertainty such as economic crisis (Liounis, 2011), natural disasters (Shareef and McAleer, 2007), or political upheaval (Hussain, 2014).

Perception of uncertainty is an important factor for consumers’ purchasing decisions (Muthukrishnan et al., 2009; Schumann et al., 2010; Van Horen and Pieters, 2013). For instance, consumers feel anxious especially when they have to assess the best products or services under uncertain conditions (Way and Jeanine Meyers, 2013). In general, consumers act to minimize their anxiety level to reach the best decision (De Vries et al., 2010), but excessive levels of uncertainty can result in biased judgments with regard to actual product performance and pricing (Gao et al., 2002).

Many studies reported cultural variation in levels of uncertainty avoidance. According to Hofstede et al.’s (2010) dataset, high uncertainty avoidance cultures include Greece (100 points), Russia (95), and Japan (92), while low uncertainty avoidance cultures include the United States (46), China (30), and Singapore (8). Researchers have further delved into detailed decision-making processes where they identify patterns of substantial cultural variations such as reliability, novelty, and tolerance toward uncertainty. Here, we briefly review representative studies of these subdomains.

#### Reliability and Uncertainty Avoidance

Previous marketing research suggests that consumers from strong uncertainty avoidance cultures prefer reputable brands and products more than those from weak uncertainty avoidance cultures. For example, Lee et al. (2007) reported that watches made in Switzerland (i.e., a country widely known for manufacturing quality watches) are more positively evaluated by consumers in strong (e.g., South American cultures) than weak uncertainty avoidance cultures (e.g., the United States, the United Kingdom, Hong Kong, and India), suggesting that the reliability of a product assures strongly uncertain consumers.

#### Novelty and Uncertainty Avoidance

Diversity in attitude toward new services is also a target of investigation using this construct. De Bellis et al. (2015) demonstrated that consumers from strong uncertainty avoidance cultures (e.g., Japan, Taiwan) were also cautious about adopting new technology. They were less likely to use retailers’ websites to customize their cars compared to those from weak uncertainty avoidance cultures (e.g., Singapore, China). Also, consumers in weak uncertainty avoidance cultures such as the United States preferred innovative smartphones more than consumers in strong uncertainty avoidance cultures such as Japan and Brazil (Leng and Botelho, 2010).

#### Tolerance and Uncertainty Avoidance

Level of tolerance is associated with uncertainty avoidance: Consumers from weak uncertainty avoidance cultures are more tolerant of ambiguity. For instance, restaurant patrons in weak uncertainty avoidance cultures such as Singapore and China do not mind changing restaurants as much as those in strong uncertainty avoidance cultures such as Germany and Taiwan (Tse and Crotts, 2005).

## Organizational Studies Relating to Culture

Organizational studies are a major area in business research that investigates human behavior in organizational settings. We believe that due to globalization, cross-cultural investigation has become indispensable. The opportunities for research are broad and include individual-level behaviors such as work motivation and decision-making; group-level behaviors such as leadership, human resource management, and teamwork; and intergroup level behaviors ranging from persuasion, to negotiation, to international business management. A plethora of cross-cultural studies have been published over the past decade (e.g., Gelfand et al., 2007; Peterson and Barreto, 2018; Tung and Stahl, 2018). In this section, we will briefly review some classic and recent findings in this area of research, which have applied at least one of the six cultural constructs to their investigations.

### Research Utilizing the Individualism vs. Collectivism Construct

#### Work Performance

Earley’s (1993) study on human behaviors in the workplace is one of the first to cross-culturally investigate organizational behavior under this cultural construct. The study examined managers’ performance in three conditions: working individually, with in-group members, and with out-group members. The results showed that Chinese and Israeli managers perform better in the in-group condition, whereas American managers perform best working individually, and that people’s subjective sense of the structure of the workplace differs depending on whether their culture is collectivistic or individualistic. This research opened new, fruitful topics such as whether cultural diversity in a group facilitates or inhibits performance or insight. The number of related investigations has increased constantly (e.g., Milliken and Martins, 1996; Keller, 2001; Shapiro et al., 2002; Page, 2007; Putnam, 2007; Stahl et al., 2010; Loyd et al., 2012; Pieterse et al., 2013; Galinsky et al., 2015; Chua, 2018). Human performance optimization is a particularly hot topic in today’s global industries.

#### Corporate Governance

This area of study focuses on the systems through which corporations’ policies and objectives are set and pursued in the context of the social, regulatory, and market environments (e.g., monitoring the actions, policies, practices, and decisions of corporations and their stakeholders (OECD, 2015). One of the most accessible topics for cultural psychologists to initiate research is on the issue of corruption. Corruption is a common and costly phenomenon, and there are clear cultural variations in how it is defined, how it manifests, and how it is dealt with. Obvious factors related to corruption include level of deception and honesty, but Heine (2012) discusses several social factors which exacerbate long-term corruption behaviors, such as poverty, economics, and power inequality. Other studies have argued that collectivism leads to excessive in-group loyalty, resulting in increased motivation to establish “cozy relationships” (e.g., Judge et al., 2011; Zheng et al., 2013; Ren et al., 2016; Jha and Panda, 2017). In another report which addressed collectivism and bad deeds in the workplace, Leung et al. (1990, 1992) demonstrate that people in collectivistic cultures are more likely than their individualistic counterparts to comply with, compromise with, and accommodate another party’s requests. Corporate governance remains a rich area for cross-cultural research.

#### Leadership

Studies in this area represent some of the earliest cross-cultural investigations, and the topics are diverse. For example, Misumi and Peterson (1985) introduced a two-axis model to analyze patterns of leadership: Performance orientation vs. Maintenance orientation, measuring the strength of balanced leadership and the weakness of leadership which falls to an extreme or lacks both. Preference for performance-oriented leadership was found to be desirable in English-speaking societies, while Latin American societies preferred maintenance-oriented leadership. In a more recent comprehensive cross-cultural investigation of leadership, House et al. (2004) and Javidan et al. (2006) classified expected types of leadership in 26 countries. The findings indicated that two types of leadership are commonly valued across cultures: charismatic/value-based leadership or team-based leadership. Charismatic/value-based leaders have the ability to inspire subordinates and value high individual performance, whereas team-based leaders value effective teamwork and set shared goals. Their findings mirror Misumi and Peterson’s classic work, finding the former leadership type is the most common in English speaking societies, and the latter is common in Latin America. Leadership studies at the company or political party level would be highly valued and represent a good area of opportunity for cultural psychologists.

### Research Utilizing the Independence vs. Interdependence Construct

This construct has been frequently used in marketing studies but is underrepresented in organizational studies. However, some early work from organizational psychology on group relations and human resource management which touches on this concept does exist.

#### Intergroup and Intra-Group Relations

The desire for the previously mentioned “guanxi”—long-term relationships based on trust and mutual benefit—and the concept of “losing face” are topics that fall into this category. Guanxi and face have been found to be desirable in interdependent societies (Yang, 1994; Fu and Yukl, 2000; Chen and Farh, 2010). There is opportunity to test these cultural values in other societies. Negotiation style is also a topic that deserves more empirical attention in today’s global world.

#### Human Resource Management

Since Hofstede’s (1980) initial investigation, international companies’ employees and their work values have been a target of cross-cultural studies. However, collected employee data is often used to merely classify national characteristics, and research on the relationships between individuals’ personal values, work ethics, and culturally dominant constructs such as self-construals remains underdeveloped. Nonetheless, some cross-cultural studies have begun to address this gap by asking how national characteristics and self-construals influence people’s intrinsic vs. extrinsic motivations (Gahan and Abeysekera, 2009). We believe that Human Resource Management holds great opportunities for cross-cultural research in social cognition, emotion, and motivation.

### Research Utilizing the Analytic vs. Holistic Cognition Construct

Due to its experiment-oriented approach, the majority of studies which utilize *analytic vs. holistic* cognition focus on basic psychological processes. Therefore, little research has been conducted in the organizational context. However, the seeds of opportunity are visible in some recent studies.

#### Persuasion

Peng and Akutsu (2001) reported that, when asked how much they agreed or disagreed with various ontological and epistemological statements, Americans were more likely to agree with linear (i.e., analytical) statements while Japanese were more likely to agree with dialectical (i.e., holistic) statements, which suggests that people’s beliefs about the nature of the world, knowledge, and human life are different between the two cultures. Peng and Akutsu (2001) argue that, due to differences in mentality, the likelihood of accepting or being persuaded by new ideas varies across cultures. A dialectical (vs. linear) mentality may facilitate a more receptive reaction to new ideas that are contradictory, ambiguous, or uncertain while it may be accompanied by a tendency to accept them at face value. Their results echo other studies’ findings on East Asians’ dialectical and holistic thinking styles: leniency to contradiction and holistic reference to all available information.

#### Leadership

Some cultural psychologists are already actively researching this topic. For instance, Miyamoto and Wilken (2010) reported that the interpersonal influence orientation, common in the United States, facilitated patterns of context-independent analytic cognition, whereas the interpersonal adjustment orientation facilitated holistic, context-dependent cognition patterns. Recently, research on the contingencies of leadership styles to respective cultures elucidated that, when subjects were primed for interpersonally accommodating leadership, both European Canadians’ and Japanese’s patterns of attention became holistic, but when they were primed for interpersonal influence leadership, only European Canadians became analytic, showing the Japanese’s robust holistic tendency. These studies could create a foundation for collaborative research between cultural and organizational psychology.

Leadership and persuasion are topics with great potential for cultural psychologists to initiate collaborative research on organizational phenomena. Specifically, cultural diversity of leadership, management style, and decision-making are some exciting avenues in which to find common ground with business scholars. We encourage development in this area.

### Research Utilizing the Horizontal vs. Vertical Orientation Construct

When one establishes an organizational structure, hierarchy and power issues between members of the organization are unfailingly taken into account. Therefore, there is an obvious affinity between horizontal vs. vertical orientation and the field of organizational behavior. Culture’s influence on the issues of hierarchy, status, and power has begun to be discussed in business research.

#### Power

Recent findings suggest that power is conceptualized in two ways: personalized power and socialized power. Personalized power refers to the endowment of power to meet one’s self-centered goals to influence others, whereas socialized power refers to the endorsement of power by means of prosocial goals to benefit others. Torelli and Shavitt (2010) discussed a strong association between vertical individualism and personalized power and horizontal collectivism and socialized power. To et al. (2017) discussed how vertical collectivism facilitates people to perceive a direct relationship between high power and high status.

#### Social Status

Recent studies demonstrate diversity in perception of social status under the framework of horizontal vs. vertical orientation. For example, Park et al. (2013) reported that there is a positive association between high status and expression of anger in Japan, a representative vertical collectivistic society. In contrast, such association is weak in the United States, a representative vertical individualistic society (see also Kowner and Wiseman, 2003). Similarly, Kuwabara et al. (2016) reported a positive association between status and punishment of others in vertical collectivistic societies such as East Asia, whereas such a combination was negatively associated in vertical individualistic societies such as the United States. While it requires further investigation, one interpretation of these findings is that vertical collectivism gives high-status individuals innate privilege over and respect from surrounding others, which in turn allows them to express negative emotion freely in public and enjoy dominance over others. However, this kind of association is weak in the United States due to its high level of individualism.

#### Intrapersonal and Interpersonal Relationships

In vertical individualistic societies such as Israel, once one gains power, it facilitates a level of egocentrism and assertiveness to claim resources, whereas such an association is weak in vertical collectivistic societies such as Hong Kong (Kopelman, 2009). Similarly, there is diversity in negotiation styles: Vertical collectivism has an association with low-power individuals’ sense of accommodation to high-power partners, while such a sense of accommodation is low in both vertical and horizontal individualistic societies (Kopelman et al., 2016).

These lines of studies provide insight into potential cross-cultural research on management, negotiation, and impression formation. Thus far, findings under the vertical and horizontal construct have been mostly based on experimental studies, but they provide cultural psychology researchers opportunities to delve into actual practices in the workplace. In this sense, the level of affinity with organizational research is promising.

### Research Utilizing the Tightness vs. Looseness Construct

Because the tightness vs. looseness construct was introduced to the field quite recently, there are limited studies in which it is used to analyze organizational behavior. However, some notable findings have been reported in the context of management as well as interpersonal relations, the majority of which are case-study-based reports.

#### Management

In Harvard Business Review, Gelfand et al. (2018) noted that the discrepancy in levels of tightness vs. looseness orientation negatively affected the Whole Foods Inc. and Amazon Inc. merger. They argued that there were clear differences in the companies’ organizational cultures. Amazon’s tight company culture values consistency and routine, is less tolerant of rebellious behavior, and has strict rules and processes to uphold the company’s traditions. In contrast, Whole Foods’ loose company culture values fluidity, shuns rules, and encourages breakthrough ideas. These two companies’ leadership also differed substantially: Amazon’s leaders emphasized independence, confidence, and top-down decision-making whereas Whole Foods was led by visionary and collaborative leaders. As implied by Gelfand et al.’s report, mismatches of organizational cultures during mergers could be an important future field of research in the intercultural context of globalization.

#### Interpersonal Relations

Gunia et al. (2011) reported that negotiators in a loose culture (e.g., the United States) develop a better sense of interpersonal trust compared to those in a tight culture (e.g., India) because interpersonal skills are necessary to establish a solid relationship without the help of institutional and normative affiliations. In contrast, negotiators in a tight culture are low in interpersonal trust because they have institutionally established affiliations, as well as their family name and their community’s reputation and partnerships, which negatively affect their motivation to create a new relationship under uncertain contexts (e.g., Tucker, 2019).

### Research Utilizing the Strong vs. Weak Uncertainty Avoidance Construct

#### Innovation

The literature suggests that weak uncertainty avoidance is associated with risk-taking behaviors. As innovation processes are perceived as uncertain and risky, acceptance of uncertainty positively influences the initiation and implementation of innovation projects. Studies have shown that uncertainty-accepting cultures (i.e., Greece, Mexico, Germany) tend to yield higher national-level innovation success (Shane, 1993) and cultivate managerial preferences for innovation (Shane, 1995). Current business pressures to adopt innovation in the face of sustainability challenges make this area lucrative for further study.

#### International Business

The literature suggests that uncertainty avoidance is associated with acceptance to change. Kogut and Singh (1988) found that firms entering high-uncertainty avoidance cultures (i.e., Belgium, France) consider acquisitions to be less attractive because integrating foreign management into the parent organization is considered riskier than direct entry to a market. As employees in uncertainty-avoiding cultures (i.e., Greece, Portugal) are expected to be less willing to accept change (Harzing and Hofstede, 1996), an acquisition may increase inefficiencies and incur additional costs while integrating. Therefore, a cooperative mode (i.e., international joint venture) or greenfield venture, both of which involve less change, is preferred.

Looking at a sample of Japanese firms which entered the Western European market, Brouthers and Brouthers (2000) reconfirmed that firms entering societies strong in uncertainty avoidance (i.e., Germany, France) tend to prefer greenfield investments. Barr and Glynn (2004) found that the concept of uncertainty avoidance is related to how individuals perceive controllability: business managers in societies strong in uncertainty avoidance (i.e., Japan, Germany) associate lack of controllability with business threat, while business managers in societies with low uncertainty avoidance (i.e., the United States, Sweden) perceive a lack of controllability as a business opportunity.

## Summary

This section introduced representative studies in the area of organizational studies. Although the range of topics is broader than that of Marketing, the number of studies utilizing the six cultural constructs is limited. This may be due to the fact that organizational behavior researchers often base their studies on a particular set of case studies of companies and organizations. In addition, even those scholars who support cultural construct-based research may deal with cultural variations in behaviors as a country-level phenomenon and avoid using the constructs at the organization or individual level, partially due to the tradition of Hofstede’s type of approach. Recently, Torelli et al. (2020) stated that individual-level analysis using psychological research methodology is gaining popularity. As business scholars shift their level of analysis from country level to individual level, further affinity between cultural psychology and business research will be established.

## Future Directions

For over 30 years, cultural psychologists have demonstrated that there are substantial cultural variations in even basic psychological processes and have advocated for theoretical advancement in psychology. Their main objective was to make psychological theories more culture-friendly, intending to depict mutual constitutions of culture and human psychology (Geertz, 1973; Bruner, 1990; Shweder, 1991; Miller, 1999). Given that business scholars set a “to do” list for future investigations, how can cultural psychologists gain entry into this new field? What can cultural psychologists contribute to the potential collaboration in research? Here, we address some responses to these questions.

### Developing More Indigenous and Emic-Oriented Constructs

Henrich et al.’s (2010) paper on WEIRD (Western Educated Industrialized Rich and Democratic) societies critiques Western dominance with regard to data sampling which biases scholars’ attitudes toward culture. In the past 30 years of development of cultural psychology, the voices from non-western individuals’ points of view have also gradually increased. Simultaneously, intercultural and inter-national collaborations have become more substantial, going far beyond requesting basic data collection of local collaborators without deep knowledge of the culture. However, there is still much room for improvement in diversifying the data to include unbiased analyses of underrepresented peoples, cultures, and societies.

The indigenous concept of *guanxi* (Yang, 1994; Fu and Yukl, 2000; Chen and Farh, 2010) is a good example of scholars’ accomplishments at broadening the field, as is the recent movement of indigenous psychology (Kim and Berry, 1993; Kim et al., 2006) that is facilitating studies targeting native peoples. For example, to capture emic constructs and merge them into etic constructs, Berry (1989) suggested five steps to integrate constructs from two different cultures: (1) examine a research problem in one’s own culture and develop a conceptual framework; (2) transport this conceptualization and measurement to examine the same issue in a similar manner in another culture; (3) enrich the imposed etic framework with unique aspects of the second culture; and examine the two sets of findings for comparability. (4) If these findings are not comparable, the two conceptualizations will be considered independent, and (5) if they are comparable, then the common set, termed as derived etic, will form the basis of a unified etic framework.

To date, however, few studies have demonstrated truly generalizable indigenous and etic constructs. One of the strengths of business research is their long tradition of case study. For example, a plethora of case studies demonstrated the uniqueness of Japanese corporate activities and ideas (e.g., Nonaka and Takeuchi, 1995), and such accumulation of culturally unique knowledge should be incorporated into our ongoing theoretical frameworks. In response to the need for more unique cultural data, our research team initiated an indigenous cultural study by targeting Mongolian school-age children and identified that their way of pictorial representation was more similar to Japanese than to European Canadians. This type of endeavor in the culture and business context will allow us to better refine the relationships between the six cultural constructs and provide more nuanced evidence of cultural diversity.

### Identifying Boundary Conditions in Combinations of Multiple Constructs

Scholars in cultural psychology have recently become aware of the importance of developing a fine-grained theoretical framework to better capture societal-level phenomena and individual-level phenomena. For example, Miyamoto (2013) addressed multilevel analysis models to better understand the relationships between existing dimensional constructs. Similarly, Varnum et al. (2010) attempted to synthesize the constructs of self-construals (independent vs. interdependent) and thinking styles (analytic vs. holistic thought).

In addition to the synthesis of theoretical frameworks, identification of boundary conditions has begun. For example, Ito et al. (2013) asked both Japanese and European Canadians to judge a target figure’s facial expression while manipulating the types of background: non-agentic scenic images vs. agentic images with figures. The results indicated that, while Japanese Canadians’ judgments were influenced by both the non-agentic and agentic backgrounds, European Canadians were influenced only when the target was exposed to non-agentic backgrounds, suggesting that European Canadians selectively ignore background information in order to separate the target’s emotion from that of others—the dimension of self-construals played a more important role than the dimension of thinking styles. Ito et al. (2013) assert that this type of investigation can help identify the boundary condition of each type of construct and specify under what conditions people in a given culture manifest particular behaviors. We expect that the number of studies using this type of investigation will increase shortly in cultural psychology, and the concreteness of activities such as price judgment, brand image judgment, and managerial behaviors which have been commonly investigated in the field of consumer and organizational psychology will contribute to disentangling the dynamic nature of cultural phenomena.

### Focusing More on Intercultural Phenomena

The spread of globalization and increasingly multicultural societies and workplaces mean that intercultural factors will likewise become increasingly important in many or most areas of research. In our desire to understand cultural differences, we have often neglected to consider how individuals are acculturated when they spend significant time in intercultural situations. For example, we have evidence that East Asian Canadians’ patterns of attention fell between European Canadians’ analytic patterns and Japanese holistic patterns (Masuda et al., 2012). In terms of the six cultural constructs, we may speculate that an Asian immigrant in a given Western society changes his/her self-construal from being interdependent to being independent as they acculturate, but still keep their holistic cognition. Intercultural settings are rich environments that can enable researchers to better map the strength of cultural constructs against human behavior. Acculturation scholars have engaged in such research, but cultural psychologists and business researchers should collaboratively include intercultural factors in investigations of international organizations. It will enrich opportunities to delve into more dynamic and complex settings regarding power and hierarchical structures. Below, we introduce several studies that make excellent jumping-off points for new research that could include such intercultural factors.

In their intercultural communication study, Ward and Kennedy (1993, 1999) address the construct “cultural distance,” by which they identified the importance of cultural matching: Malaysian international students showed better cultural adaptation when they studied in culturally similar Singapore compared to culturally distal New Zealand. Due to globalization, people increasingly move from one culture to another and experience culture shock. The greater the cultural distance, the greater the shock. The issue of cultural distance is also examined in consumer psychology. For example, De Bellis et al. (2019) demonstrated that customers are motivated to purchase when target products are presented with culturally fit forms; the use of the interfaces that match consumers’ thinking styles (analytic vs. holistic) leads to greater subjective ease, which in turn generates positive consumer responses such as enhanced product satisfaction and higher purchase likelihood.

Similarly, De Bellis et al. (2015) demonstrated that, when international customers have access to a customization interface they are not familiar with when purchasing a car, prevention-oriented Japanese and Taiwanese customers tended to withdraw from the procedure, whereas promotion-oriented Chinese and Singaporean customers accepted the uncertainty and tended to complete the procedure. By targeting concrete decision-making activities, cultural psychologists can receive great benefit from delving into human activities in cultural contexts.

As society is becoming increasingly globalized and connected, we expect more research avenues for topics related to the interactions or mixing of culture. Cultural mixing occurs when symbolic representations from different cultures are simultaneously available in the same place (Chiu et al., 2009). Scholars taking a dynamic constructivist approach to culture acknowledge that cultures are understood as interactive and continuously evolving systems and that different elements of culture can coexist within one individual, situation, or environment (Hong et al., 2000; Hao et al., 2016).

According to Chiu et al. (2011), when people are exposed to mixed cultures, two types of responses occur: exclusionary response, which is a defensive process evoked against the threat of loss of one’s own culture, or integrative response, which is a mental process of understanding, accepting, and combining a foreign culture with one’s own culture, facilitating creativity and the regeneration of multiple cultures. Studying consumer reactions to cultural mixing, Peng and Xie (2016) found that divergent consequences (i.e., exclusion or integration) occur when people focus on either differences or similarities between two cultures. In three studies, they found that American and Chinese participants who were primed with the difference between Chinese and Western cultures (e.g., food, architecture, brands) evaluate culture mixing products (e.g., a mouse pad or luxury watch) less preferably than those who were primed with the similarity between the two cultures. Studies focusing on the positive outcomes of culture mixing explore how multicultural individuals straddle different cultural schemas or switch into different cultural codes to create work-related outcomes (Molinsky, 2007). For example, multicultural individuals are known to develop intercultural skills that help them to be responsive and/or be aware of cultural cues in the diverse work context (Earley and Peterson, 2004) and perform better at creative tasks (Godart et al., 2015).

### Refining Methodology

To date, the majority of dimensional approaches have been content with survey methodology—using a scale and an assorted battery of questionnaires, or testing a mediational factor to explain the relationship between culture and particular behaviors. While such findings provide us a variety of insights, further methodological development is expected. Fortunately, social and cultural psychologists have devised a variety of methodologies such as priming, situation sampling, perceptual, and cognitive methodologies using behavioral tasks (Masuda et al., 2019 for review). Furthermore, the advent of cultural neuroscience (Han and Northoff, 2008; Kitayama and Uskul, 2011) allows scholars to elucidate the cultural origins of brain activities and can provide insightful ideas not only to psychology but to a wide range of fields including consumer and organizational psychology. We expect that the number of neuroscientific studies will drastically increase in consumer and organizational psychology in the next 10 years.

## Conclusion

Now, the time has come to initiate greater interdisciplinary collaboration between cultural, organizational, and consumer psychology. There are plenty of benefits for cultural psychologists who get involved in new collaborations (1) as we can access individuals who engage in concrete decision-making in daily contexts, which enriches the quality of investigation; (2) furthermore, the concreteness helps cultural psychologists to experience insights which are different from those we usually experience in laboratories with relatively abstract experimental stimuli; (3) ultimately, this opportunity is a chance for cultural psychologists to interact with scholars in experimental economics and other social sciences.

Before we close the paper, we would like to give readers a tip: one might think that a substantial paradigm shift or a theoretical breakthrough is necessary when one conducts an interdisciplinary study. But actually, the change need not be drastic. Bit by bit advances based on previous findings make the possibility of collaboration more realistic and more substantial. For example, Masuda et al. (2019) investigated whether their findings of cultural variation in perception of emotion can be generalizable in a workplace setting, with affirmative results from their first sample. As such, like a diesel locomotive, it will be slow to start, but constant and patient accumulation of empirical findings will allow us to pull many freight cars to new research horizons.

## Author Contributions

TM and KI mainly developed the ideas and wrote the manuscript. YY, JL, SS, and SA provided professional information, and partially contributed to the writing of the manuscript. All authors contributed to the article and approved the submitted version.

## Conflict of Interest

The authors declare that the research was conducted in the absence of any commercial or financial relationships that could be construed as a potential conflict of interest.
